# Multi-Body-Site Microbiome and Culture Profiling of Military Trainees Suffering from Skin and Soft Tissue Infections at Fort Benning, Georgia

**DOI:** 10.1128/mSphere.00232-16

**Published:** 2016-10-05

**Authors:** Jatinder Singh, Ryan C. Johnson, Carey D. Schlett, Emad M. Elassal, Katrina B. Crawford, Deepika Mor, Jeffrey B. Lanier, Natasha N. Law, William A. Walters, Nimfa Teneza-Mora, Jason W. Bennett, Eric R. Hall, Eugene V. Millar, Michael W. Ellis, D. Scott Merrell

**Affiliations:** a Department of Microbiology and Immunology, Uniformed Services University of the Health Sciences, Bethesda, Maryland, USA; b Infectious Disease Clinical Research Program, Department of Preventive Medicine and Biostatistics, Uniformed Services University of the Health Sciences, Bethesda, Maryland, USA; c Henry M. Jackson Foundation for the Advancement of Military Medicine, Rockville, Maryland, USA; d Department of Medicine, Uniformed Services University of the Health Sciences, Bethesda, Maryland, USA; e Martin Army Community Hospital, Fort Benning, Georgia, USA; f Department of Microbiome Science, Max Planck Institute for Developmental Biology, Tübingen, Germany; g Infectious Diseases Directorate, Wound Infections Department, Naval Medical Research Center, Silver Spring, Maryland, USA; h Walter Reed Army Institute of Research, Silver Spring, Maryland, USA; i The University of Toledo Medical Center, Toledo, Ohio, USA; University at Buffalo

**Keywords:** MRSA, microbiome, SSTI, *Staphylococcus aureus*, USA300

## Abstract

Skin and soft tissue infections (SSTIs) are common in the general population, with increased prevalence among military trainees. Previous research has revealed numerous nasal microbial signatures that correlate with SSTI development and *Staphylococcus aureus* colonization. Thus, we hypothesized that the ecology of the inguinal, oropharynx, and perianal regions may also be altered in response to SSTI and/or *S. aureus* colonization. We collected body site samples from 46 military trainees with purulent abscess (SSTI group) as well as from 66 asymptomatic controls (non-SSTI group). We also collected abscess cavity samples to assess the microbial composition of these infections. Samples were analyzed by culture, and the microbial communities were characterized by high-throughput sequencing. We found that the nasal, inguinal, and perianal regions were similar in microbial composition and significantly differed from the oropharynx. We also observed differences in *Anaerococcus* and *Streptococcus* abundance between the SSTI and non-SSTI groups for the nasal and oropharyngeal regions, respectively. Furthermore, we detected community membership differences between the SSTI and non-SSTI groups for the nasal and inguinal sites. Compared to that of the other regions, the microbial compositions of the nares of *S. aureus* carriers and noncarriers were dramatically different; we noted an inverse correlation between the presence of *Corynebacterium* and the presence of *Staphylococcus* in the nares. This correlation was also observed for the inguinal region. Culture analysis revealed elevated methicillin-resistant *S. aureus* (MRSA) colonization levels for the SSTI group in the nasal and inguinal body sites. Together, these data suggest significant microbial variability in patients with SSTI as well as between *S. aureus* carriers and noncarriers.

**IMPORTANCE** While it is evident that nasal colonization with *S. aureus* increases the likelihood of SSTI, there is a significant lack of information regarding the contribution of extranasal colonization to the overall risk of a subsequent SSTI. Furthermore, the impact of *S. aureus* colonization on bacterial community composition outside the nasal microbiota is unclear. Thus, this report represents the first investigation that utilized both culture and high-throughput sequencing techniques to analyze microbial dysbiosis at multiple body sites of healthy and diseased/colonized individuals. The results described here may be useful in the design of future methodologies to treat and prevent SSTIs.

## Abstract (precis)

While it is evident that nasal colonization with *S. aureus* increases the likelihood of SSTI, there is a significant lack of information regarding the contribution of extranasal colonization to the overall risk of a subsequent SSTI. Furthermore, the impact of *S. aureus* colonization on bacterial community composition outside the nasal microbiota is unclear. Thus, this report represents the first investigation that utilized both culture and high-throughput sequencing techniques to analyze microbial dysbiosis at multiple body sites of healthy and diseased/colonized individuals. The results described here may be useful in the design of future methodologies to treat and prevent SSTIs.

## INTRODUCTION

Skin and soft tissue infections (SSTIs) encompass a wide array of skin maladies that range in clinical presentation from mild (e.g., abscess or cellulitis) to severe (e.g., necrotizing fasciitis). Despite the variation in presentation, the bulk of disease manifests as purulent abscess and cellulitis. For military service members, especially trainees, SSTIs are common and may even prevent successful completion of training (1, 2). High rates of infection are believed to be due to close living conditions, high frequency of skin abrasions, and imperfect hygiene practices that foster an environment conducive to colonization, transmission, and infection with *Staphylococcus aureus* (3–8). Indeed, *S. aureus* has routinely been identified as the most common cause of cutaneous abscesses (9, 10). In the general community, *S. aureus*-associated SSTIs have become a serious health concern in the United States (11–13), and their treatment is costly and challenging. This is especially true with the emergence of strains of methicillin-resistant *S. aureus* (MRSA) (11, 12). To combat SSTIs, previous strategies have aimed to prevent or clear *S. aureus* colonization (14–17). Unfortunately, the majority of these studies have mixed results, suggesting that other microbe- and host-related factors may also contribute to SSTI risk and therefore warrant further investigation. Study of the interaction of microbial communities (microbiota) with their respective human hosts has revolutionized modern medicine (18, 19). Thus, we set out to understand the associations between SSTI, *S. aureus* colonization, and microbial composition in military trainees.

Given the high percentage (20% to 40%) of healthy individuals in the United States who are nasal carriers of *S. aureus*, most colonization studies have largely concentrated on the anterior nares (20–22). Indeed, these reports have demonstrated the impact of SSTI development as well as *S. aureus* colonization on nasal microbial composition (22, 23). Importantly, nasal carriers of *S. aureus*, and especially MRSA, are significantly more likely to develop SSTI than noncarriers (24, 25). However, in spite of extensive research, it is still unclear why some nasal carriers of *S. aureus* develop SSTI while others do not. We now appreciate that *S. aureus* colonizes multiple body sites, including the oropharynx, inguinal, and perianal regions (26–28). However, the contribution of *S. aureus* extranasal colonization to SSTI risk has not been well characterized. Therefore, we hypothesized that, in addition to the nose, the microbiota at other body sites may differ among individuals with and without SSTI and/or between *S. aureus* carriers and noncarriers. To address this hypothesis, we enrolled military trainees that either did or did not have a purulent abscess and collected colonization swabs from multiple body sites. Specimens were analyzed using culture and high-throughput sequencing. In an effort to improve current SSTI treatment and prevention measures, we sought to reveal important microbiological signatures throughout the body that may correlate with SSTI and colonization with *S. aureus*.

## RESULTS

### Participant characteristics and *Staphylococcus aureus* carriage.

In total, we enrolled 112 military trainees, 46 (41%) of whom presented with a purulent abscess (SSTI group). Sixty-six trainees were enrolled as healthy controls (non-SSTI group). Baseline characteristics of the study participants, including *S. aureus* colonization prevalence at multiple body sites, are outlined in Table 1. The two study groups were similar in age (*P* = 1.00) and race/ethnicity (*P* = 0.8) (Table 1). *S. aureus* colonization status at four sites (nose, oropharynx, inguinal, perianal) was assessed for all participants. Of the 112 participants, 84 (75.0%) provided samples from all four body sites. In total, we obtained 108 nasal, 108 oropharynx, 94 inguinal, and 89 perianal culture samples. Seventy-six (67.9%) participants had *S. aureus* (MRSA or methicillin-sensitive *S. aureus* [MSSA]) cultured from at least one body site (76.1% versus 62.1% for SSTI versus non-SSTI groups, respectively). While the prevalences of MSSA and no *S. aureus* (NoSA) colonization at the various body sites were comparable between groups, MRSA colonization prevalence was more variable (Table 1); MRSA colonization prevalence was higher among SSTI than non-SSTI participants, particularly in the nasal (19.0% versus 13.6%) and inguinal (14.3% versus 7.7%) regions. Additionally, of the 40 abscesses for which we obtained culture results, the majority were *S. aureus* positive (50.0% MRSA, 45.5% MSSA). Of these, 36 were characterized by pulsed-field type (PFT); USA300 (66.7%) was the most common PFT followed by USA200 (5.6%), USA400 (5.6%), and USA1000 (2.8%). Seven isolates (19.4%) had patterns that did not correspond to any known PFT (see Table S1 in the supplemental material). In addition to the abscess isolates, we found that the majority of MRSA strains isolated from the other four body sites were USA300 (see Table S1). While numerous MSSA USA300 strains were also isolated throughout the body, there were a significant number of MSSA strains that did not correspond to any known PFT (see Table S1). Other PFTs (USA200, USA400, US500, USA600, USA700, USA800, USA1000) were less frequently isolated from the various body sites (see Table S1).

10.1128/mSphere.00232-16.5 Table S1  PFGE typing of sample body sites and abscesses. Download Table S1, DOCX file, 0.1 MB. Copyright © 2016 Singh et al. 2016 Singh et al. This content is distributed under the terms of the Creative Commons Attribution 4.0 International license.

**TABLE 1:** Patient characteristics^d^

Parameter	Values (%)^a^		*P* value^b^
	SSTI group (*n* = 46)	Non-SSTI group (*n* = 66)	
Age			
Median age, yrs (range)	20 (18–28)	20 (18–30)	1.00
			
Race/ethnicity			
White, non-Hispanic	34 (73.9)	47 (71.2)	0.80
Hispanic	8 (17.4)	11 (16.7)	
Black, non-Hispanic	2 (4.3)	6 (9.1)	
Other, non-Hispanic	2 (4.3)	2 (3.0)	
			
Nasal colonization			
MRSA	8 (19.0)	9 (13.6)	0.75
MSSA	17 (40.5)	28 (42.4)	
NoSA	17 (40.5)	29 (43.9)	
			
Oropharynx colonization			
MRSA	2 (4.4)	3 (4.8)	0.93
MSSA	21 (46.7)	27 (42.9)	
NoSA	22 (48.9)	33 (52.4)	
			
Inguinal colonization			
MRSA	6 (14.3)	4 (7.7)	0.55
MSSA^c^	15 (35.7)	18 (34.6)	
NoSA	21 (50.0)	30 (57.7)	
			
Perianal colonization			
MRSA	4 (9.5)	4 (8.5)	0.90
MSSA	15 (35.7)	15 (31.9)	
NoSA	23 (54.8)	28 (59.6)	
			
Abscess colonization			
MRSA	20 (50.0)	NA	
MSSA	18 (45.0)	NA	
NoSA	2 (5.0)	NA	
			
Site of infection			
Lower extremity	21 (45.7)	NA	
Knee	7 (15.2)		
Thigh	6 (13.0)		
Foot	4 (8.7)		
Lower leg	2 (4.3)		
Buttock	2 (4.3)		
Upper extremity	11 (23.9)	NA	
Forearm	7 (15.2)		
Elbow	2 (4.3)		
Arm	1 (2.2)		
Hand	1 (2.2)		
Thorax	11 (23.9)	NA	
Axilla	9 (19.6)		
Abdominal	1 (2.2)		
Chest	1 (2.2)		
Head and neck	3 (6.5)	NA	
Face	2 (4.3)		
Neck	1 (2.2)		

a Unless otherwise specified, all values represent the number of total individuals in each group followed by the percentage in parentheses. Samples with missing data for a specified characteristic were removed in calculating percentages.

b *P* values were computed using the chi-square statistical test.

c At the time of analysis, antibiotic susceptibility data for one *S. aureus* isolate were not available. The isolate was conservatively labeled as MSSA.

d Abbreviations: MRSA, methicillin-resistant *Staphylococcus aureus*; MSSA, methicillin-sensitive *Staphylococcus aureus*; NA, not applicable; NoSA, no *Staphylococcus aureus*.

Multi-body-site colonization data revealed that when *S. aureus* (MRSA or MSSA) was isolated from any single body site, there was a significant chance that *S. aureus* would also be isolated from one or more additional body sites (*P* < 0.05) (see Fig. S1A in the supplemental material). Furthermore, this phenomenon was also observed when MRSA or MSSA data were analyzed separately (see Fig. S1B and C). The same was true for coagulase-negative staphylococcus (CNS), the other major group for which culture data were available (see Fig. S1D). When the purulent abscess culture data were analyzed in tandem, we found strain concordance between the inguinal region and abscess specimens; when MRSA or MSSA was cultured from the inguinal body site, MRSA or MSSA was also isolated from the abscess, and vice versa (*P* < 0.05) (see Fig. S1B and C).

10.1128/mSphere.00232-16.1 Figure S1  Colonization concordance among the various body sites for *S. aureus* (MRSA and MSSA) (A), MRSA (B), MSSA (C), and coagulase-negative staphylococcus (Coag-Staph) (D). For each cell, the large number corresponds to the percentage of individuals for which the colonization parameters were true. The fraction in each cell shows the breakdown of that percentage. The more red the cell, the larger the percentage. Each graph is to be read from top to bottom and then from left to right. For example, in panel A, for those individuals that had *S. aureus* in their anterior nares (59 total), 74.6% (44 total) of them also had *S. aureus* in their oropharynx. For those individuals that did not have *S. aureus* in their anterior nares (45 total), 17.8% (8 total) of them had *S. aureus* isolated from the oropharynx. To determine if a given result was statistically significant, we performed chi-square analysis for each pair of cells. For comparisons with low numbers of counts, the Fisher exact test was used. Cell pairs shown with a slash through them were not significant (*P* > 0.05). All other comparisons were statistically significant (*P* < 0.05). Download Figure S1, TIF file, 2.6 MB. Copyright © 2016 Singh et al. 2016 Singh et al. This content is distributed under the terms of the Creative Commons Attribution 4.0 International license.

We also observed a similar trend with respect to PFT USA300 (see Fig. S2 in the supplemental material). If USA300 was isolated from one body site, there was a significant chance it would also be isolated from one or more additional body sites (*P* < 0.05) (see Fig. S2C). This was true with respect to consideration of MRSA USA300 or MSSA USA300 independently (see Fig. S2A and B). However, with respect to abscess isolates, we found that oropharyngeal colonization with USA300 did not coincide with USA300 presence in the abscess (see Fig. S2C); this was true even when MRSA and MSSA were considered individually (see Fig. S2A and B). Similarly to the oropharynx, perianal colonization with MRSA USA300 did not coincide with the presence of USA300 in the abscess (see Fig. S2A).

10.1128/mSphere.00232-16.2 Figure S2  Colonization concordance among the various body sites for *S. aureus* (MRSA USA300) (A), MSSA USA300 (B), and total USA300 (C). For each cell, the large number corresponds to the percentage of individuals for which the colonization parameters were true. The fraction in each cell shows the breakdown of that percentage. The more red the cell, the larger the percentage. Each graph is to be read from top to bottom and then from left to right. For example, in panel A, for those individuals that had MRSA USA300 in their anterior nares (12 total), 25.0% (3 total) of them also had MRSA USA300 in their oropharynx. For those individuals that did not have MRSA USA300 in their anterior nares (92 total), 0.0% (0 total) of them had MRSA USA300 isolated from the oropharynx. To determine if a given result was statistically significant, we performed chi-square analysis for each pair of cells. For comparisons with low numbers of counts, the Fisher exact test was used. Cell pairs with a slash through them were not significant (*P* > 0.05). All other comparisons were statistically significant (*P* < 0.05). Download Figure S2, TIF file, 1.7 MB. Copyright © 2016 Singh et al. 2016 Singh et al. This content is distributed under the terms of the Creative Commons Attribution 4.0 International license.

### Sequencing results.

Body site samples from the 112 participants (458 samples total) were sequenced over four separate sequencing platform (MiSeq) reactions (114 to 115 samples per run). In total, we obtained 110 nasal, 109 oropharynx, 97 inguinal, 96 perianal, and 46 abscess microbiome samples. Sequencing of the 458 samples yielded a total of 58,490,530 raw sequences. Of these, 28,322,655 (48.4%) of the sequences remained after quality filtering and contaminate removal. In total, each sample had approximately 61,840 associated reads (range, 5,174 to 167,518), with an average read length of 433 bp (range, 374 to 475). Prior to diversity analyses, 5,714 reads were randomly subsampled from each sample. Both Good’s coverage values (>99%) and rarefaction curves (data not shown) suggested that the subsampled data accurately depicted the total microbiota within each sample. Percent abundance data for all microbiome samples are included in Table S2 in the supplemental material.

10.1128/mSphere.00232-16.6 Table S2  Percent abundance of each phylotype per sample according to the Ribosomal Database Project (RDP) database. Each column represents one sample and is labeled on top with a specific ID number (four-digit ID number followed by body location indicated as follows: A, abscess; N, nose; O, oropharynx; I, inguinal; P, perianal). Sample ID numbers that do not end with an “R” represent individuals who had a purulent abscess (SSTI group). ID numbers that end in an “R” represent individuals who were part of the non-SSTI control group. The total number of reads associated with each sample is listed at the bottom of each column. The rank ID column represents the taxonomic rank for each phylotype (e.g., 0.1.1 = phylum level, 0.1.1.1 = class level, 0.1.1.1.1 = order level, 0.1.1.1.1.1 = family level, 0.1.1.1.1.1.1 = genus level). Download Table S2, XLSX file, 2.8 MB. Copyright © 2016 Singh et al. 2016 Singh et al. This content is distributed under the terms of the Creative Commons Attribution 4.0 International license.

### Multi-body-site microbiota characterization.

**FIG 1:**
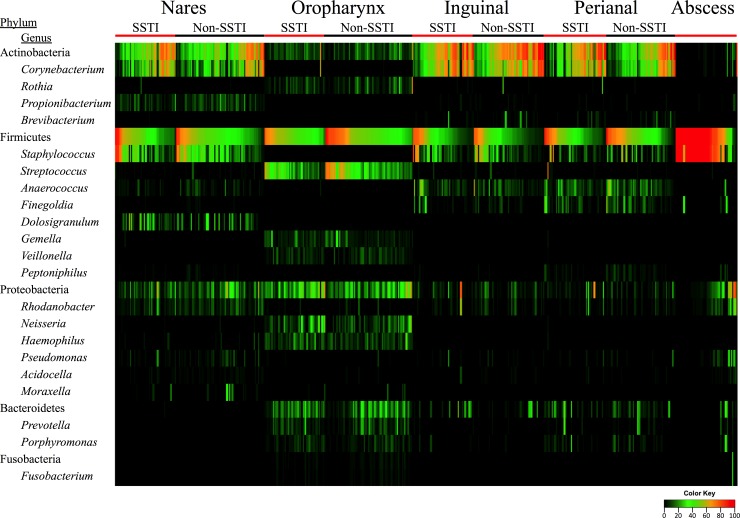
Heat map showing the percent abundance of the predominant phyla and genera for the five body sites tested. Within each body site, the samples are separated according to SSTI status (SSTI and non-SSTI). Each column corresponds to a single sample. Percent abundance values are described in the color key.

Microbial profiling of the various body sites revealed that the nasal, inguinal, and perianal samples shared numerous microbial signatures, including a high abundance of *Actinobacteria* and *Firmicutes* (Fig. 1) (Table 2). Furthermore, a large proportion (50% to 70%) of the samples from these three body sites were predominantly comprised of only two genera: *Corynebacterium* and *Staphylococcus* (Fig. 1) (Table 2). However, unlike the other regions, the oropharynx had lower levels of *Actinobacteria*, with elevated levels of *Proteobacteria*. *Streptococcus* represented the most abundant genus, with trace amounts of *Staphylococcus* seen.

**TABLE 2:** Representative body site microbiota abundance levels

Phylotype^a^	Phylotype abundance^b^				
	Nose	Oropharynx	Inguinal	Perianal	Abscess
***Actinobacteria***	44.63	10.93	59.45	47.04	3.96
* Corynebacterium*	32.26	0.62	51.90	39.22	3.31
* Rothia*	0.22	6.69	0.58	0.55	0.01
* Propionibacterium*	6.75	<0.01	0.37	0.30	3.36
* Brevibacterium*	0.03	<0.01	1.61	1.77	<0.01
					
***Firmicutes***	41.97	49.33	31.56	42.03	79.05
* Staphylococcus*	28.51	0.01	17.11	9.56	72.54
* Streptococcus*	0.59	31.32	0.42	1.02	0.13
* Anaerococcus*	2.41	0.05	8.31	10.87	1.20
* Finegoldia*	0.30	<0.01	2.48	6.84	2.51
* Dolosigranulum*	8.85	<0.01	<0.01	<0.01	<0.01
* Gemella*	0.09	5.87	<0.01	0.05	<0.01
* Veillonella*	0.03	4.56	<0.01	0.03	<0.01
* Peptoniphilus*	0.50	<0.01	0.22	1.79	0.81
					
***Proteobacteria***	12.76	23.39	4.92	4.88	12.81
* Rhodanobacter*	5.73	0.77	2.49	1.93	6.88
* Neisseria*	0.15	10.86	<0.01	0.04	<0.01
* Haemophilus*	0.58	8.34	0.01	0.09	0.01
* Pseudomonas*	2.02	0.21	0.73	0.87	3.36
* Acidocella*	0.89	0.11	0.40	0.31	0.94
* Moraxella*	1.38	0.55	0.01	0.01	<0.01
					
***Bacteroidetes***	0.22	13.17	3.69	5.37	3.21
* Prevotella*	0.03	6.09	0.17	1.60	0.88
* Porphyromonas*	0.04	3.73	0.34	2.16	2.18
					
***Fusobacteria***	<0.01	1.07	<0.01	0.01	0.84
* Fusobacterium*	<0.01	0.83	<0.01	0.01	0.82

a Phylum-level phylotypes are shown in bold and italics. Genus-level phylotypes are shown in italics.

b Values represent average phylotype abundance levels for all participants.

**FIG 2:**
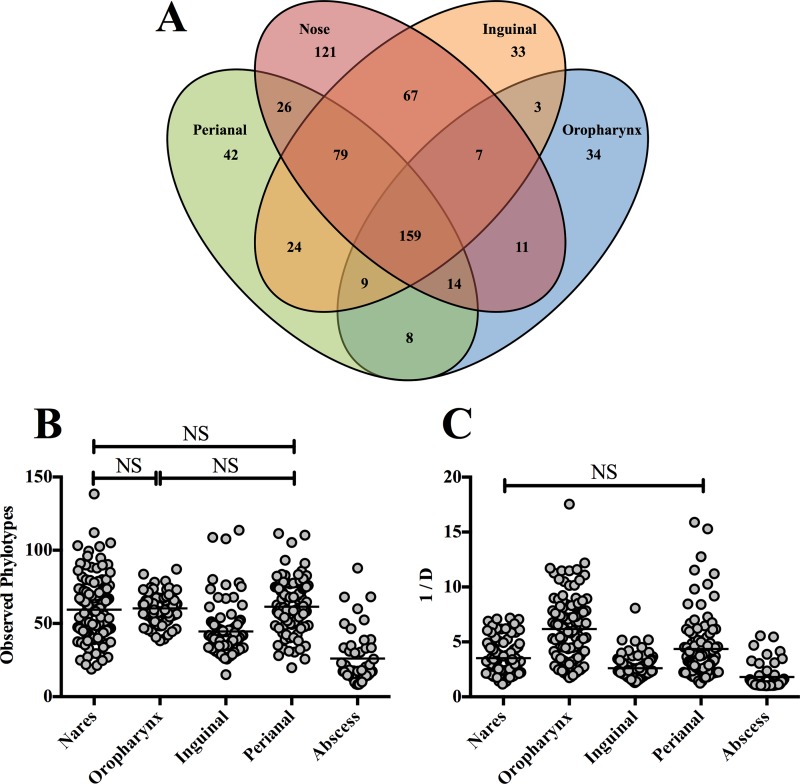
Phylotype distribution, richness, and diversity levels for the various body sites and abscesses. (A) Venn diagram showing the number of phylotypes at each body site. Values within overlapping circles represent phylotypes shared among those body sites. (B) Phylotype richness as assessed by the mean number of observed phylotypes per sample. (C) Sample diversity levels as determined using the invsimpson index (1/D). The higher the value, the more diverse the sample. For panels B and C, each circle represents a single sample and the mean values are indicated by a solid black line. All comparisons were statistically significant (*P* < 0.001) except for those marked with an NS (not significant).

With respect to bacterial diversity, the nose harbored the highest number of phylotypes (484), 121 of which were not detected at any other body site (Fig. 2A). Conversely, the oropharynx harbored the lowest number of phylotypes (245). Despite the differences in phylotypes, the bacterial richness in the nose (average number of phylotypes per sample, 59.47) was not significantly different from that in the oropharynx (average number of phylotypes per sample, 60.34) (*P* > 0.05) and perianal (average number of phylotypes per sample, 61.42) (*P* > 0.05) regions (Fig. 2B). However, the inguinal body site was significantly reduced in phylotype richness (*P* < 0.0001) (Fig. 2B). When overall diversity was assessed using the inverse Simpson (invsimpson) index, the phylotypes in the inguinal body site were again the least diverse (*P* ≤ 0.0002) (Fig. 2C) among the four regions. The nares and perianal body sites were comparable in levels of bacterial diversity (*P* > 0.05), with the oropharynx representing the most diverse body site examined (*P* < 0.0001) (Fig. 2C).

**FIG 3:**
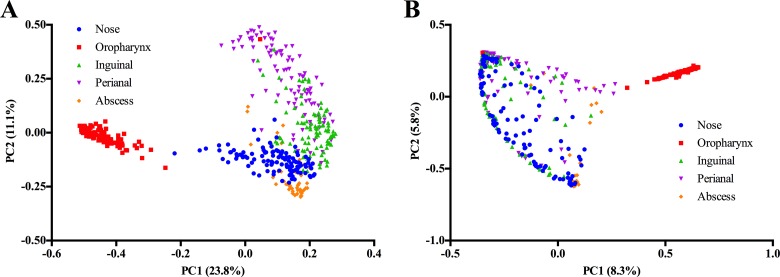
Principal-coordinate analysis (PCoA) showing the variation in body site microbiomes as determined using the Jaccard (A) and Θ_YC_ (B) diversity calculators. Each colored symbol corresponds to an individual sample. The variation represented by each axis (PC1 or PC2) is shown in parentheses.

Beta-diversity analyses further emphasized the unique microbial composition of the oropharynx compared to the other three body sites as determined by the Θ_YC_ and Jaccard metrics (Fig. 3; see also Fig. S3 in the supplemental material). Principal-coordinate analysis (PCoA) of the Θ_YC_ and Jaccard distance matrices revealed clear separation of the oropharynx samples from the other regions (Fig. 3). Of note, unlike the other regions, the oropharynx samples appeared to be highly conserved in bacterial membership (see Fig. S3A) as determined by the Jaccard metric.

10.1128/mSphere.00232-16.3 Figure S3  Heat map comparison of the various body site samples to each other as determined by the Jaccard (A) and Θ_YC_ (B) diversity calculators. Each comparison results in a value from 0 to 1, where 0 (red) represents high similarity and 1 (black) represents low similarity. The color key is shown below the heat maps. Download Figure S3, TIF file, 2.7 MB. Copyright © 2016 Singh et al. 2016 Singh et al. This content is distributed under the terms of the Creative Commons Attribution 4.0 International license.

### Microbial differences between SSTI and non-SSTI participants.

**FIG 4:**
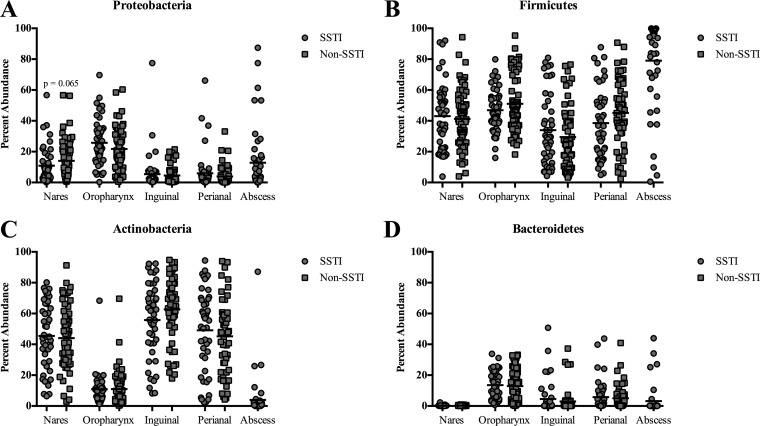
Percent abundance of the various bacteria phyla at the 4 body sites and abscesses. The data for each body site are broken into individuals who either had SSTI (circles) or did not have SSTI (non-SSTI) (squares). Each symbol corresponds to an individual sample. The black line represents the mean abundance. For *Proteobacteria* in the nares, the *P* value shown was generated using the Mann-Whitney statistical test.

**FIG 5:**
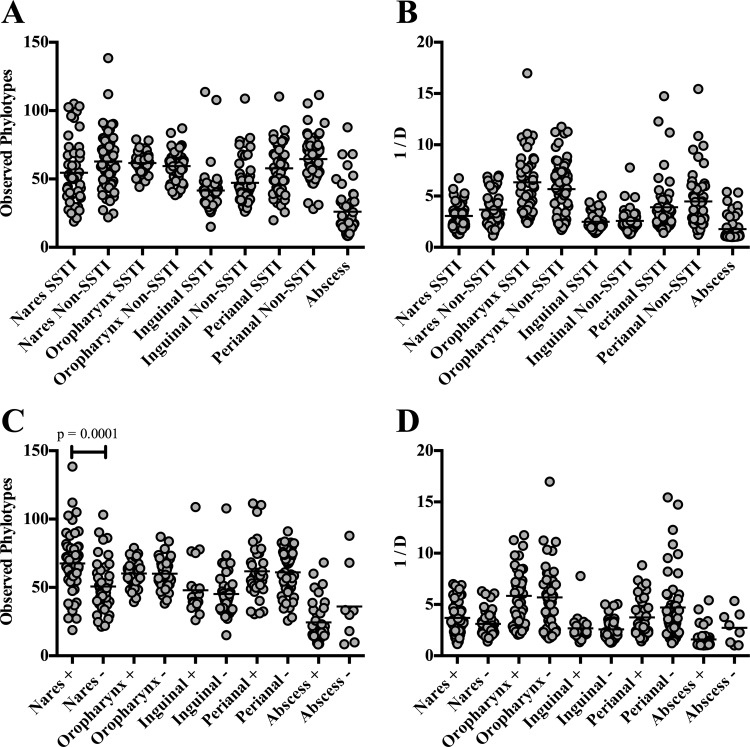
Microbiome comparisons for the various body sites between those that had an SSTI and those that did not (non-SSTI) (A and B) and between those body sites that were culture positive and those that were negative for *Staphylococcus aureus* (C and D). We assessed phylotype richness (observed phylotypes) (A and C) as well as overall diversity using the inverse Simpson index (1/D) (B and D). Each symbol corresponds to an individual sample. The black line represents the mean abundance. Means that were statistically different from each other between SSTI and non-SSTI or *S. aureus* positive (+) or negative (−) samples were determined using ANOVA. Significant *P* values (*P* < 0.05) are indicated on the graphs.

We observed a trend toward a decreased prevalence of *Proteobacteria* in the nares of military trainees with SSTI compared to non-SSTI controls (*P* = 0.065) (Fig. 1 and 4). The percentages of abundance of the other phyla did not significantly differ between the SSTI and non-SSTI participants (Fig. 4). Furthermore, there were no detectable differences in the number of observed phylotypes per sample or in diversity levels (invsimpson) between the SSTI and non-SSTI groups for each region (Fig. 5A and B).

We next conducted Metatstats analysis in order to assess phylotype abundance differences between the SSTI and non-SSTI groups at the various body sites. The nose and oropharynx were the only two regions for which we observed significant differences in phylotype abundance (Table 3). Individuals with SSTI had a significantly lower level of *Anaerococcus* (1.4% versus 3.1%, *P* = 0.016) and *Streptococcus* (27.1% versus 34.4%, *P* = 0.033) in their nares and oropharynx, respectively, than the non-SSTI patients (Table 3). We also found that SSTI patients had a higher level of *Granulicatella* in their oropharynx than the members of the non-SSTI group (2.6% versus 1.1%, *P* = 0.002) (Table 3).

**TABLE 3:** Metastats analysis

Body site	Phylotype^a^	Mean percent abundance		Metastats *P* value^b^
		Community 1	Community 2	
Nose	*Anaerococcus*	SSTI (1.4)	Non-SSTI (3.1)	0.016
Nose	*Corynebacterium*	*S. aureus*^+^ (17.5)	*S. aureus*^−^ (47.3)	<0.001
Nose	*Staphylococcus*	*S. aureus*^+^ (40.6)	*S. aureus*^−^ (17.1)	<0.001
Nose	*Dolosigranulum*	*S. aureus*^+^ (5.5)	*S. aureus*^−^ (11.9)	0.021
Nose	*Propionibacterium*	*S. aureus*^+^ (8.2)	*S. aureus*^−^ (5.1)	0.016
Nose	*Rhodanobacter*	*S. aureus*^+^ (6.7)	*S. aureus*^−^ (4.4)	0.045
Nose	*Pseudomonas*	*S. aureus*^+^ (3.3)	*S. aureus*^−^ (0.8)	0.004
Nose	*Acidocella*	*S. aureus*^+^ (1.1)	*S. aureus*^−^ (0.7)	0.042
Oropharynx	*Streptococcus*	SSTI (27.1)	Non-SSTI (34.4)	0.033
Oropharynx	*Granulicatella*	SSTI (2.6)	Non-SSTI (1.1)	0.002
Oropharynx	*Gemella*	*S. aureus*^+^ (4.5)	*S. aureus*^−^ (7.7)	0.014
Inguinal	*Staphylococcus*	*S. aureus*^+^ (11.7)	*S. aureus*^−^ (20.4)	0.027
Perianal	*Clostridiales*; unclassified family	*S. aureus*^+^ (1.3)	*S. aureus*^−^ (2.5)	0.028
Perianal	*Roseburia*	*S. aureus*^+^ (0.3)	*S. aureus*^−^ (1.2)	0.022

a According to the Ribosomal Database Project (RDP) database.

b Only data from significant (*P* < 0.05) comparisons are shown.

To determine if these abundance differences had an effect on community structure, we generated *P* values using analysis of molecular variance (AMOVA) on the Jaccard and Θ_YC_ distance matrices. We detected a difference in phylotype membership between the SSTI and non-SSTI groups for the nasal and inguinal body sites (Jaccard AMOVA, *P* = 0.02 and 0.018, respectively) (Table 4). Despite differences in membership, with respect to phylotype abundance, all body sites were indistinguishable between the SSTI and non-SSTI groups (Θ_YC_ AMOVA, *P* > 0.05) (Table 4). The oropharynx data, however, showed a trend toward significance in the AMOVA test of the Θ_YC_ distance matrix (*P* = 0.055), which suggests a potential difference in oropharynx community structure between the SSTI and non-SSTI participants.

**TABLE 4:** Beta-diversity comparisons between communities

Body site	Comparison groups		Jaccard *P* value^a^	Θ_YC_ *P* value^a^
	Community 1	Community 2		
Nose	SSTI	Non-SSTI	**0.020**	0.204
				
Nose	*S. aureus*^+^	*S. aureus*^−^	**<0.001**	**<0.001**
				
Nose	SSTI and *S. aureus*^+^	SSTI and *S. aureus*^−^	**0.006**	**<0.001**
				
Nose	Non-SSTI and *S. aureus*^+^	Non-SSTI and *S. aureus*^−^	**<0.001**	**<0.001**
				
Oropharynx	SSTI	Non-SSTI	0.135	0.055
				
Oropharynx	*S. aureus*^+^	*S. aureus*^−^	0.368	0.101
				
Oropharynx	SSTI and *S. aureus*^+^	SSTI and *S. aureus*^−^	0.566	0.188
				
Oropharynx	Non-SSTI and *S. aureus*^+^	Non-SSTI and *S. aureus*^−^	0.188	**0.011**
				
Inguinal	SSTI	Non-SSTI	**0.018**	0.554
				
Inguinal	*S. aureus*^+^	*S. aureus*^−^	0.700	0.064
				
Inguinal	SSTI and *S. aureus*^+^	SSTI and *S. aureus*^−^	0.846	0.234
				
Inguinal	Non-SSTI and *S. aureus*^+^	Non-SSTI and *S. aureus*^−^	0.320	0.308
				
Perianal	SSTI	Non-SSTI	0.097	0.340
				
Perianal	*S. aureus*^+^	*S. aureus*^−^	0.172	0.465
				
Perianal	SSTI and *S. aureus*^+^	SSTI and *S. aureus*^−^	0.338	0.220
				
Perianal	Non-SSTI and *S. aureus*^+^	Non-SSTI and *S. aureus*^−^	0.183	0.578
				
Abscess	MRSA	MSSA	0.242	0.211
				
Abscess	MRSA	NoSA	**0.043**	**<0.001**
				
Abscess	MSSA	NoSA	**0.017**	**0.001**

a *P* values are the result of performing analysis of molecular variance (AMOVA) on the Jaccard and Θ_YC_ distance matrices. Significant *P* values (<0.05) are shown in bold.

### *S. aureus* impact on microbial composition.

**FIG 6:**
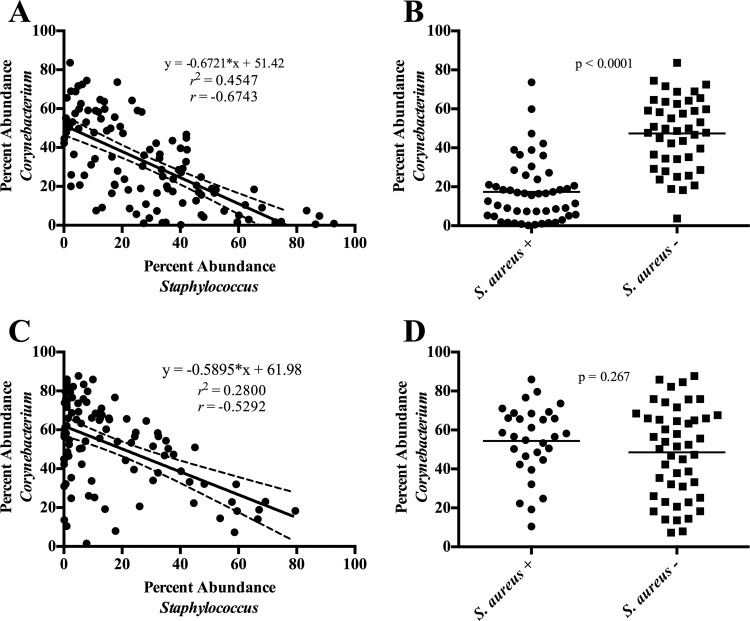
Abundance of *Corynebacterium* compared to the abundance of *Staphylococcus* (A and C) and *Staphylococcus aureus* (B and D) for the nasal (A and B) and inguinal (C and D) body sites. Each symbol corresponds to an individual nasal or inguinal sample. For panels A and C, solid lines and inset equations represent the line of best fit [*y* = slope(*x*) + *y* intercept]. Dashed lines represent the 95% confidence interval. *r*^2^, coefficient of determination; *r*, correlation coefficient. For panels B and D, the *S. aureus* designation was determined by culture. Solid lines correspond to the mean percent abundance. *P* values shown were generated using the Mann-Whitney statistical test.

Because we did not detect any significant differences in community structure at any region between MRSA-colonized and MSSA-colonized individuals as determined using AMOVA on the Θ_YC_ distances (*P* > 0.05), we subsequently classified regions as either *S. aureus* (MRSA and MSSA) positive or negative (NoSA). The microbial compositions of the nares of *S. aureus*-positive and *S. aureus*-negative communities were dramatically different from those of the other regions tested. Indeed, while the overall diversity levels did not significantly differ (*P* > 0.05) (Fig. 5D), *S. aureus*-positive nasal communities were found to be significantly richer in diversity than *S. aureus*-negative nasal communities (*P* = 0.0001) (Fig. 5C). Statistical AMOVA of the Jaccard (*P* < 0.001) and Θ_YC_ (*P* < 0.001) distance matrices revealed significant alterations in bacterial membership and abundance levels between *S. aureus*-positive and *S. aureus*-negative nasal communities (Table 4). Furthermore, Metastats analysis revealed multiple bacterial genera that differed significantly in abundance levels between the two groups, including *Corynebacterium*, *Staphylococcus*, and *Dolosigranulum* (Table 3). Of note, levels of *Staphylococcus* and *Corynebacterium* were inversely correlated in the nares (coefficient of determination [*r*^2^] = 0.4547, correlation coefficient [*r*] = −0.6743, slope [*m*] = −0.6721, *P* < 0.0001) (Fig. 6A). Furthermore, the average percent abundance of *Corynebacterium* in the nares of *S. aureus*-negative communities was significantly greater than that of *S. aureus*-positive individuals (*P* < 0.0001) (Fig. 6B).

Outside the nares, we also observed a significant inverse correlation between *Staphylococcus* and *Corynebacterium* at the inguinal region (coefficient of determination [*r*^2^] = 0.2800, correlation coefficient [*r*] = −0.5292, slope [*m*] = −0.5895, *P* < 0.0001) (Fig. 6C). However, the overall levels of *Corynebacterium* among *S. aureus*-positive and -negative individuals were similar (*P* = 0.267) (Fig. 6D). Additionally, total *Staphylococcus* abundance was reduced in *S. aureus*-positive individuals compared to *S. aureus*-negative individuals (11.7% versus 20.4%, Metastats, *P* = 0.027) (Table 3).

Intragroup variation in microbial composition between *S. aureus* carriers and noncarriers for the SSTI group as well as the non-SSTI group was also assessed. *S. aureus* nasal carriage status was associated with a significant difference in microbial composition for both the SSTI and non-SSTI groups (Jaccard and Θ_YC_ AMOVA, *P* ≤ 0.006) (Table 4). For the oropharynx, however, we observed a difference in the Θ_YC_ distances between the *S. aureus*-positive and *S. aureus*-negative individuals for the non-SSTI group only (*P* = 0.011) (Table 4). There were no differences in microbial composition between *S. aureus* carriers and noncarriers within the SSTI and non-SSTI groups at the inguinal and perianal regions (Jaccard and Θ_YC_ AMOVA, *P* > 0.05).

Overall, with the exception of the oropharynx, we observed high levels of *Staphylococcus* at the various regions. To evaluate if the abundance levels at one body site correlated with the abundance levels at other body sites, we performed linear regression analysis and found a positive correlation for the *Staphylococcus* abundance levels between the inguinal and perianal regions (coefficient of determination [*r*^2^] = 0.2385, correlation coefficient [*r*] = 0.4884, slope [*m*] = 0.3902, *P* < 0.0001) (see Fig. S4 in the supplemental material).

10.1128/mSphere.00232-16.4 Figure S4  Linear regression analysis comparing the abundance levels of *Staphylococcus* at the perianal and inguinal body sites. Each symbol corresponds to an individual participant. The solid lines and inset equation represent the line of best fit [*y* = slope(*x*) + *y* intercept]. Dashed lines represent the 95% confidence interval. *r*^2^, coefficient of determination; *r*, correlation coefficient. Download Figure S4, TIF file, 0.3 MB. Copyright © 2016 Singh et al. 2016 Singh et al. This content is distributed under the terms of the Creative Commons Attribution 4.0 International license.

### The abscess microbiota.

Sequencing analysis of the 46 purulent abscess samples revealed that *Firmicutes* was the dominant phylum present (average abundance, 79.1%) (Fig. 1). While *Staphylococcus* dominated the vast majority of abscess samples (average abundance, 72.5%), we observed a significant number of polymicrobial infections; 22 of the 46 (47.8%) samples had no single genus with over 90% abundance (Fig. 1 and Table 2). The polymicrobial abscesses were frequently inhabited by bacteria from the genera *Rhodanobacter*, *Pseudomonas*, and *Corynebacterium* (average abundances, 6.9%, 3.4%, and 3.3%, respectively) (Table 2).

Given that most of the bacterial populations of the abscesses were composed of a single phylotype (*Staphylococcus*), we observed a remarkably low level of phylotype richness and diversity (observed phylotypes and invsimpson) compared to the other regions (Fig. 2A and B). Subsequently, we observed a high level of homogeneity between abscess samples in phylotype membership (Jaccard) and abundance (Θ_YC_) (Fig. 3; see also Fig. S3 in the supplemental material). When we subcategorized the abscesses based on *S. aureus* culture results, we found no significant differences in the observed phylotypes and invsimpson values between *S. aureus*-positive and *S. aureus*-negative abscesses (*P* > 0.05) (Fig. 5C and D). However, beta diversity comparisons revealed significant differences in phylotype membership and abundance between the MRSA and NoSA abscesses (Jaccard AMOVA, *P* = 0.043; Θ_YC_ AMOVA, *P* < 0.001), as well as between the MSSA and NoSA abscesses (Jaccard AMOVA, *P* = 0.017; Θ_YC_ AMOVA, *P* = 0.001) (Table 4).

## DISCUSSION

Previous studies have revealed that antecedent nasal colonization with *S. aureus*, especially MRSA, is a notable risk factor for SSTI (29–31). Furthermore, studies of the nasal microbiota have shown that the presence of *S. aureus* can dramatically impact the microbial community composition (22, 23, 32). However, no study has assessed the potential associated microbial dysbiosis at extranasal regions. Therefore, we set out to assess the impact of *S. aureus* colonization and/or SSTI at multiple anatomic regions (nose, oropharynx, inguinal, perianal) among military trainees known to be at increased risk for SSTI. To our knowledge, our study was the first of its kind to utilize both culture and high-throughput sequencing techniques to evaluate the relationships among infection, colonization, and microbiome with respect to *S. aureus*, a major cause of SSTI in both hospital- and community-based settings. Together, these findings highlight a dynamic interplay of microbial factors, some of which may contribute to an individual’s risk of SSTI when exposed to and subsequently colonized with *S. aureus*.

We found that while each site tested harbored a unique microbial composition (Fig. 3), the nasal, inguinal, and perianal regions shared numerous microbial signatures (Fig. 1). In contrast, the oropharynx not only was unique in its microbial composition (Fig. 1) but was significantly more diverse than any of the other regions tested (Fig. 2). Despite these differences, a common feature shared among all body sites was the ability to culture *S. aureus*. Thus, it is interesting to speculate about how *S. aureus* behaves in these variable microbial communities. In particular, how can the interaction of *S. aureus* with different bacterial cohabitants ultimately influence the overall abundance levels of *S. aureus*? Moreover, could the expression profile of *S. aureus* at the various body sites make it more or less “primed” to cause infection? These and other SSTI microbiome-related questions are under investigation in our laboratory.

The nasal microbiota of the SSTI and non-SSTI participants were clearly different. Interestingly, the microbial dysbiosis between the SSTI and non-SSTI groups in the nose was greater than that seen for any of the other tested regions (Fig. 3 and 5 and Tables 3 and 4). Additionally, we observed a difference in phylotype membership between the SSTI and non-SSTI nasal communities (Table 4). While it is difficult to speculate on the role of these differences with respect to risk of SSTI, we do note that these findings corroborate our previous nasal microbiota study conducted among military trainees (22). This represents a validation of our prior observations and further supports a link between nasal microbiota composition and SSTI.

Other than the anterior nares, the only body site for which we identified differences in bacterial abundance levels between the SSTI and non-SSTI groups was the oropharynx (Table 3); trainees with abscesses had lower levels of *Streptococcus* than the trainees in the non-SSTI groups (Table 3). With respect to the potentially protective role of *Streptococcus* against SSTI formation, previous reports suggested that *Streptococcus pneumoniae* can inhibit the growth of *S. aureus* (33, 34). Therefore, increased levels of *Streptococcus* in the oropharynx may decrease the risk of SSTI by inhibiting the acquisition and/or growth of *S. aureus*.

Besides *Streptococcus*, there is significant interest regarding the interaction of *Corynebacterium* with *S. aureus*. It has been demonstrated that various species of *Corynebacterium* can either inhibit or promote the growth of *S. aureus* (32). Indeed, previous studies have observed an inverse correlation between the levels of *S. aureus* and *Corynebacterium* in the anterior nares (22, 32, 35, 36). This inverse correlation was even stronger with respect to the entire *Staphylococcus* genus. Here, we also observed an inverse correlation between *Staphylococcus* and *Corynebacterium* (Fig. 6A). Furthermore, there was significantly more *Corynebacterium* bacteria in the nares of *S. aureus* noncarriers than in those of carriers (Fig. 6B). This apparent competition between *S. aureus* and *Corynebacterium* has been well documented and is likely of clinical significance given that nasal colonization with *S. aureus* often precedes SSTI formation (29–31).

The nose was not the only body site in which *Staphylococcus* and *Corynebacterium* abundance levels were inversely correlated. We observed a similar inverse correlation between these two genera in the inguinal region (Fig. 6C). However, there were comparable levels of *Corynebacterium* in the *S. aureus*-positive and *S. aureus*-negative groups on the basis of inguinal *S. aureus* colonization results (Fig. 6D)*.* Thus, the influence of *Corynebacterium* on *Staphylococcus* abundance in the inguinal body site may extend beyond just *S. aureus*. Indeed, there is evidence that *Staphylococcus epidermidis* abundance levels are also inversely correlated with *Corynebacterium* abundance (22). It is possible that *Corynebacterium* may serve as a semiuniversal “good guy” protecting against possible pathogens within the human microbiome. However, the underlying molecular mechanism of this apparent bacterial antagonism in the nasal and inguinal body sites remains unclear. Elucidation of these mechanisms is critical for better understanding of the complex dynamics between SSTI formation, *S. aureus* colonization, and the human microbiome. Moreover, these mechanisms may yield insight for the development of novel therapeutics.

Colonization with *S. aureus* alone can dramatically impact the composition of the nasal microbiota (Fig. 5 and Tables 3 and 4). Surprisingly, this effect was not observed at any of the other regions tested (oropharynx, inguinal, or perianal). Although there are undoubtedly numerous factors that may contribute to this nose-specific phenomenon, one obvious environmental difference that distinguishes the nose from the other tested body sites is temperature. While the inguinal, oropharynx, and perianal body sites reside at or around body temperature (37°C), the anterior nares are exposed to substantially lower temperatures (23 to 25°C) (37). Perhaps, upon exposure to lower temperatures, *S. aureus* activates and/or represses a distinct repertoire of genes that encode secreted factors and/or surface-exposed proteins that directly affect abundance levels of neighboring bacteria in the nasal cavity. Indeed, there have been numerous studies that indicated the presence of temperature-influenced regulons in *S. aureus*, including those associated with biofilm formation and toxin production (38, 39). Thus, the factors that govern the impact of *S. aureus* on nasal microbiota composition warrant further investigation.

In conjunction with our microbiome studies, we utilized basic culture and strain characterization to determine the *S. aureus* (MRSA, MSSA, or NoSA) colonization status for each consenting trainee in our study. With respect to the nose, we found that while the numbers of *S. aureus* (MRSA or MSSA)-colonized participants in the SSTI and non-SSTI groups were comparable (~60% for each group), the percentage of MRSA-colonized participants was higher in the SSTI group than in the non-SSTI group (Table 1). This finding is in agreement with previous studies that found a correlation between MRSA nasal colonization and SSTI development (25, 29, 40). Despite the clinical significance of nasal colonization, it is now evident that nonnasal (oropharynx, inguinal, and perianal) colonization with *S. aureus* is more common than previously recognized. Indeed, our data suggest that when *S. aureus* is isolated from any one body site, there is a high likelihood that it can also be isolated elsewhere on the body (see Fig. S1 and S2 in the supplemental material). Thus, there is significant interest in understanding if *S. aureus* colonization throughout the body is linked to overall SSTI risk. Interestingly, we found that, in addition to the colonization of the nose, inguinal colonization with MRSA was substantially more common in the SSTI group than in the non-SSTI group (Table 1). Furthermore, there was a significant likelihood that when MRSA or MSSA was isolated from the abscess, MRSA or MSSA would also be isolated from the inguinal body site (see Fig. S1 and S2). This paradigm-shifting observation suggests a potential link between the inguinal region and risk of SSTI. Of note, given the all-male study population, whether this link would also apply to females remains unknown. Additionally, considering the four regions together, it is possible that *S. aureus* colonization at body sites in frequent contact with the hands or other items that facilitate spread (i.e., towels) increases the likelihood of self-inoculation and subsequent SSTI. Although this is speculative, future studies aimed at SSTI prevention should be cognizant of *S. aureus* colonization in not only the anterior nares but the inguinal region as well.

Besides the four regions sampled, we also utilized culture and high-throughput sequencing to characterize the microbial composition of purulent abscesses. Surprisingly, while half of the abscesses were dominated by *Staphylococcus*, about half of the abscesses were polymicrobial (Fig. 1). On the basis of our current and previous (22) abscess microbiota results, we estimate that approximately 1 in every 3 purulent abscesses at Fort Benning is polymicrobial in nature. While the clinical significance of polymicrobial infections has been briefly reported (41, 42), there are likely countless variables that are impacted, including SSTI resolution time, responsiveness to antibiotics, treatment options, likelihood of severe complications (i.e., deep-tissue infection), and *S. aureus* gene expression/virulence. Many of these variables are under investigation in our laboratory.

There were limitations to our study. As with our previous nasal microbiota investigation, the current analysis represents only a snapshot of the microbiota at the various body sites. Thus, it is impossible to distinguish between *S. aureus* carriers, noncarriers, and intermittent carriers. Indeed, these various designations have been shown to carry unique microbial signatures, especially in the anterior nares (32). Thus, a multi-body-site longitudinal study that characterizes microbial communities over time is needed. Additionally, while our study investigated numerous body sites, we acknowledge that other regions of the human body (e.g., axilla) may harbor clues regarding SSTI formation. Also, our study population was composed entirely of young, healthy, male military trainees in good physical condition and therefore may not reflect the relationship between colonization and SSTIs in the general community. We also must acknowledge a potential drawback of the statistical approach regarding the multi-body-site colonization data (see Fig. S1 and S2 in the supplemental material). Use of multiple 2-by-2 chi-square tests without adjustment may inflate the type 1 error rate, resulting in some false-positive results. However, the 2-by-2 chi-square test seems most appropriate, since an overall chi-square test is not feasible given its reliance on independent observations. Overall, we note that the numbers and patterns of significant results are sufficient to suggest that the general findings of the 2-by-2 chi-square tests are true even if some of the individual significant results may represent type 1 errors. Finally, we note limitations with our sequencing platform (MiSeq) and strategy (phylotype-based) analyses. Because the forward and reverse MiSeq reads did not completely overlap, an operational taxonomic unit (OTU)-based approach was computationally impossible (43). This phylotype strategy also limited our taxonomic resolution; we were unable to reach species-level classification of the reads. Despite these limitations, our genus-level results were largely in agreement with previous body site microbiota analyses, including our own (22, 44, 45).

In conclusion, we implemented a whole-body approach to understand the links between the human microbiome, *S. aureus* colonization, and SSTI in military trainees. By utilizing a two-pronged culture and microbiome approach, we revealed numerous microbial signatures that differed between SSTI and non-SSTI groups, as well as between *S. aureus* carriers and noncarriers. We also confirmed previous findings (22) that demonstrated the monomicrobial as well as polymicrobial makeup of purulent abscesses. Together, these data provide valuable information that should prove useful in the future design of SSTI countermeasures in the military and in the general population.

## MATERIALS AND METHODS

### Study participants and design.

This cross-sectional observational study was conducted from July 2012 to December 2014. The study participants were U.S. Army soldiers undergoing 14 weeks of Infantry training at Fort Benning, GA. The study population used here ranged in age from 18 to 30 years, was in good general health, was all male, and was ethnically diverse. This study was approved by the Uniformed Services University Infectious Diseases Institutional Review Board (IDCRP-074).

### Enrollment and data collection.

Study participants were cross-sectionally identified and enrolled. Infantry trainees that sought medical care at the Troop Medical Clinic (TMC) for purulent abscess (SSTI group) or reported for a noninfectious condition, e.g., musculoskeletal complaint (non-SSTI/asymptomatic controls), were eligible for participation in the study. After written informed consent was provided, body site swab samples were obtained along with related information from the clinical microbiology laboratory and the electronic medical records. Participants underwent swab sampling (BD BBL CultureSwabs [BD Diagnostic, Sparks, MD]) at the following sites: the anterior nares, oropharynx, inguinal, and perianal regions. Participants could decline sampling of any site. Two swabs were collected from each body site, one for microbiological analysis and the other for microbiome characterization. No participants had received antimicrobials prior to sampling, with two exceptions: study identification (ID) numbers 1098 and 1411. Participant 1098 had received trimethoprim-sulfamethoxazole (TMP-SMX) 2 days prior to sampling, and participant 1411 had received doxycycline 1 day prior to sampling. Microbiome analysis of these 2 participants showed no obvious abnormalities compared to the other patients (data not shown). Exclusion criteria included the following: suspected or documented bacteremia, suspected or documented sepsis, neutropenia, chronic cellulitis, vascular insufficiency, deep soft tissue infection, surgical site infection, diabetic foot ulcers, animal or human bite wound, and infection involving the genitals.

### Swab collection protocol.

The two swabs collected from each body sites were either immediately sent for microbiological culture or placed at −80°C until microbiome analysis. Prior to incision of the abscess, the infection site was cleaned with 4% chlorhexidine and isopropyl alcohol. Abscess swab samples were collected from within the abscess cavity. The samples from the anterior nares were collected as previously described (22). Briefly, a swab was introduced 1 cm deep into a nostril and rubbed a minimum of three times in a circular manner along the nasal septum and the superior, lateral, and inferior surfaces of the nostril. The same swab was then used to sample the other nostril. A second nasal swab was then obtained following the same procedure. For the oropharynx samples, while avoiding the tongue, a swab was inserted into the mouth and rubbed gently across the tonsillar area using a combination of twirling and circular motions. The swab was rotated at least three times to ensure that the entire swab surface contacted the tonsillar mucosa. Inguinal swab samples were self-collected. Briefly, participants were instructed to hold the swab by the cap and gently rub in a twirling, circular motion, along the inguinal crease from the proximal to distal end. They were also instructed to ensure that at least three rotations of the swab were performed on each side of the groin. Perianal samples were also self-collected. Participants were instructed to handle the sterile swab by the cap and to rub it gently, with at least three rotations, across the surface of the anus, ensuring that that the entire swab head had come in contact with the anus.

### Isolate characterization.

Within 12 h of sample collection, the swabs were used to inoculate tryptic soy broth (BBL; BD Diagnostic, Sparks, MD), supplemented with 6.5% NaCl, at the Martin Army Community Hospital Microbiology Laboratory (46). Isolates of *S. aureus* were typed using pulsed-field gel electrophoresis (PFGE) and their virulence/resistance factors were evaluated using PCR as described previously (46).

### DNA extraction.

Total genomic DNA from the sample swabs was isolated using a GenElute bacterial genomic DNA kit from Sigma-Aldrich as previously described (22). Briefly, swab heads were submerged in 500 µl of Gram-positive lysis solution containing mutanolysin (125 U/ml), lysostaphin (0.16 mg/ml), and lysozyme (45 mg/ml) in a 1.5-ml Eppendorf tube and incubated at 37°C for 30 min. Proteinase K (0.95 mg/ml) and 500 µl of lysis solution C were then added to the sample, and the reaction mixture was incubated at 55°C for 10 min. Subsequent genomic DNA isolation steps were performed according to the manufacturer’s recommendation.

### DNA amplification and sequencing.

The V3-V4 region of the 16S rRNA gene was amplified from each sample using the following custom-designed primers: 338F (5′-GCCCARACWCCTACVGG-3′) and 806R (5′-GTGGACTACYVGGGTAT-3′). The reverse PCR primer contained a 12-nucleotide error-correcting Golay bar code to facilitate multiplexing of samples (47). All PCRs were carried out in a final volume of 20 µl using high-fidelity Accuprime *Taq* DNA polymerase (Invitrogen). The reaction mixture consisted of 1× Accuprime PCR buffer II, a 0.2 µM concentration of each primer, 15 µl of genomic DNA, and 0.15 µl of Accuprime polymerase (22). PCR amplification was performed in triplicate for each sample. PCR cycling conditions were in accordance with the method of Caporaso et al., with slight modification (47). Briefly, DNA was denatured at 94°C for 3 min, followed by 35 amplification cycles (94°C for 45 s, 53°C for 60 s, and 72°C for 90 s) and a final extension cycle of 10 min at 72°C. The PCR products were analyzed on a 1% agarose gel, pooled, and cleaned using a MinElute Reaction cleanup kit (Qiagen), per the manufacturer’s instructions. Purified PCR amplicons were quantified using a NanoDrop spectrophotometer (NanoDrop 8000; Thermo Scientific). Equimolar ratios of amplicons from each sample were then combined into a single sample. This combined sample was sent to the Tufts University Genomics Core Facility for paired-end 300-bp sequencing using an Illumina MiSeq platform. In total, 4 separate MiSeq runs using either 114 or 115 samples per run were performed. To obviate any sequencing bias, samples from each of the body sites (nasal, oropharynx, inguinal, and perianal) were included in each MiSeq run. Similarly, abscess samples were interspersed among 3 of the 4 MiSeq runs.

### Sequence processing and analysis.

DNA sequences were processed using mothur (v.1.36.1) in accordance with the MiSeq standard operating procedure (http://www.mothur.org/wiki/MiSeq_SOP) (48). For each of the four sequencing runs, the paired-end reads were initially merged into contigs. Contigs were discarded if they contained any ambiguous calls, were greater than 500 bp in length, or contained a homopolymer greater than 8 bp in length. Filtered sequences were aligned to the SILVA rRNA database (49). Sequences within 4 bp of each other were then merged. At this point, data from the 4 individual sequencing runs were combined and analyzed as a single data set. PCR chimeras were detected and removed from the study via mothur’s implementation of UCHIME (50). Sequences were assigned taxonomy information using the Bayesian classifier provided by the Ribosomal Database Project (RDP) and an 80% bootstrap cutoff value of over 100 iterations (51). DNA reads classified as chloroplast, mitochondria, archaea, *Eukaryota*, or “unknown” at the kingdom level were discarded. Taxonomy information was then used to bin sequences into phylotypes at the genus level. Lastly, to ensure comparability between samples for diversity analyses, 5,714 reads were randomly subsampled from each sample.

### Diversity analyses and statistics.

Diversity analyses were conducted in a fashion similar to that previously described (22). Briefly, individual sample diversity (alpha diversity) values, as well as diversity comparisons between samples (beta diversity), were calculated using mothur. The inverse Simpson (invsimpson) index was used for alpha diversity analyses, while the Jaccard and Θ_YC_ beta diversity calculators were implemented to assess similarities in phylotype membership and abundance (52, 53). Differences in microbial composition between groups (for example, SSTI versus non-SSTI) were computed using analysis of molecular variance (AMOVA) of the Jaccard and Θ_YC_ distance matrices and visualized in two dimensions using principal-coordinate analysis (PCoA). Differences in invsimpson values between groups were identified using analysis of variance (ANOVA) performed on log-transformed data followed by Tukey’s *post hoc* test for multiple comparisons. Statistical testing for percent abundance data was performed using the Mann-Whitney test. Phylotype abundance differences between groups were identified using mothur’s implementation of Metastats (54). Phylotypes that differed in abundance had to meet two criteria to be considered significant: a Metastats *P* value of less than 0.05, and a mean abundance of greater than 1% in at least one group. We utilized the 2-by-2 chi-square test to assess the likelihood of multi-body-site colonization with *S. aureus*. Comparisons with small sample sizes were corrected for by the use of the Fisher exact test. Finally, linear regression analyses were used to determine if the relationship between two variables resulted in a regression line that was significantly nonzero. Additionally, correlation coefficients were computed for all linear regression data. All statistical analyses were calculated using GraphPad Prism, with the exception of the mothur-generated AMOVA values.
