# Supplementary material for: Multi-Body-Site Microbiome and Culture Profiling of Military Trainees Suffering from Skin and Soft Tissue Infections at Fort Benning, Georgia
Source: mSphere. 2016 Oct 5;1(5):e00232-16. doi: 10.1128/mSphere.00232-16 (PMC5064451; doi:10.1128/mSphere.00232-16)
Supplement: Table S1 [file sph005162157st5.docx]

| **Table S1.** PFGE typing of sample body sites and abscesses. | | | |
| --- | --- | --- | --- |
| **Body Site / PFGE Type^a^** | **MRSA^b^** | **MSSA^b^** | **Total^b^** |
| **Nose** | 17 (27.42) | 45 (72.58) | 62 (100.00) |
| USA200 | 0 (0.00) | 9 (14.52) | 9 (14.52) |
| USA300 | 14 (22.58) | 7 (11.29) | 21 (33.87) |
| USA400 | 0 (0.00) | 2 (3.23) | 2 (3.23) |
| USA500 | 0 (0.00) | 0 (0.00) | 0 (0.00) |
| USA600 | 0 (0.00) | 2 (3.23) | 2 (3.23) |
| USA700 | 1 (1.61) | 3 (4.84) | 4 (6.45) |
| USA800 | 0 (0.00) | 2 (3.23) | 2 (3.23) |
| USA1000 | 1 (1.61) | 0 (0.00) | 1 (1.61) |
| None | 1 (1.61) | 20 (32.26) | 21 (33.87) |
| **Oropharynx** | 5 (9.43) | 48 (90.57) | 53 (100.00) |
| USA200 | 0 (0.00) | 5 (9.43) | 5 (9.43) |
| USA300 | 3 (5.66) | 7 (13.21) | 10 (18.87) |
| USA400 | 0 (0.00) | 4 (7.55) | 4 (7.55) |
| USA500 | 0 (0.00) | 1 (1.89) | 1 (1.89) |
| USA600 | 0 (0.00) | 3 (5.66) | 3 (5.66) |
| USA700 | 1 (1.89) | 4 (7.55) | 5 (9.43) |
| USA800 | 0 (0.00) | 1 (1.89) | 1 (1.89) |
| USA1000 | 1 (1.89) | 1 (1.89) | 2 (3.77) |
| None | 0 (0.00) | 22 (41.51) | 22 (41.51) |
| **Inguinal^c^** | 10 (23.81) | 32 (76.19) | 42 (100.00) |
| USA200 | 0 (0.00) | 2 (4.76) | 2 (4.76) |
| USA300 | 9 (21.43) | 8 (19.05) | 17 (40.48) |
| USA400 | 0 (0.00) | 2 (4.76) | 2 (4.76) |
| USA500 | 0 (0.00) | 0 (0.00) | 0 (0.00) |
| USA600 | 0 (0.00) | 1 (2.38) | 1 (2.38) |
| USA700 | 0 (0.00) | 3 (7.14) | 3 (7.14) |
| USA800 | 0 (0.00) | 2 (4.76) | 2 (4.76) |
| USA1000 | 1 (2.38) | 0 (0.00) | 1 (2.38) |
| None | 0 (0.00) | 14 (33.33) | 14 (33.33) |
| **Perianal** | 8 (21.05) | 30 (78.95) | 38 (100.00) |
| USA200 | 0 (0.00) | 1 (2.63) | 1 (2.63) |
| USA300 | 6 (15.79) | 8 (21.05) | 14 (36.84) |
| USA400 | 0 (0.00) | 0 (0.00) | 0 (0.00) |
| USA500 | 0 (0.00) | 0 (0.00) | 0 (0.00) |
| USA600 | 0 (0.00) | 0 (0.00) | 0 (0.00) |
| USA700 | 1 (2.63) | 6 (15.79) | 7 (18.42) |
| USA800 | 0 (0.00) | 2 (5.26) | 2 (5.26) |
| USA1000 | 1 (2.63) | 0 (0.00) | 1 (2.63) |
| None | 0 (0.00) | 13 (34.21) | 13 (34.21) |
| **Abscess** | 19 (52.78) | 17 (47.22) | 36 (100.00) |
| USA200 | 0 (0.00) | 2 (5.56) | 2 (5.56) |
| USA300 | 18 (50.00) | 6 (16.67) | 24 (66.67) |
| USA400 | 0 (0.00) | 2 (5.56) | 2 (5.56) |
| USA500 | 0 (0.00) | 0 (0.00) | 0 (0.00) |
| USA600 | 0 (0.00) | 0 (0.00) | 0 (0.00) |
| USA700 | 0 (0.00) | 0 (0.00) | 0 (0.00) |
| USA800 | 0 (0.00) | 0 (0.00) | 0 (0.00) |
| USA1000 | 1 (2.78) | 0 (0.00) | 1 (2.78) |
| None | 0 (0.00) | 7 (19.44) | 7 (19.44) |
| ^a^ None: PFGE profile could not be determined  ^b^ All values represent the number of MRSA and/or MSSA isolates at each body site followed by the percentage of the total (MRSA + MSSA) in parentheses  ^c^ At the time of analysis, PFGE data was not available for one MSSA isolate. | | | |
