# Supplementary figures and images for: Multi-Body-Site Microbiome and Culture Profiling of Military Trainees Suffering from Skin and Soft Tissue Infections at Fort Benning, Georgia
Source: mSphere. 2016 Oct 5;1(5):e00232-16. doi: 10.1128/mSphere.00232-16 (PMC5064451; doi:10.1128/mSphere.00232-16)

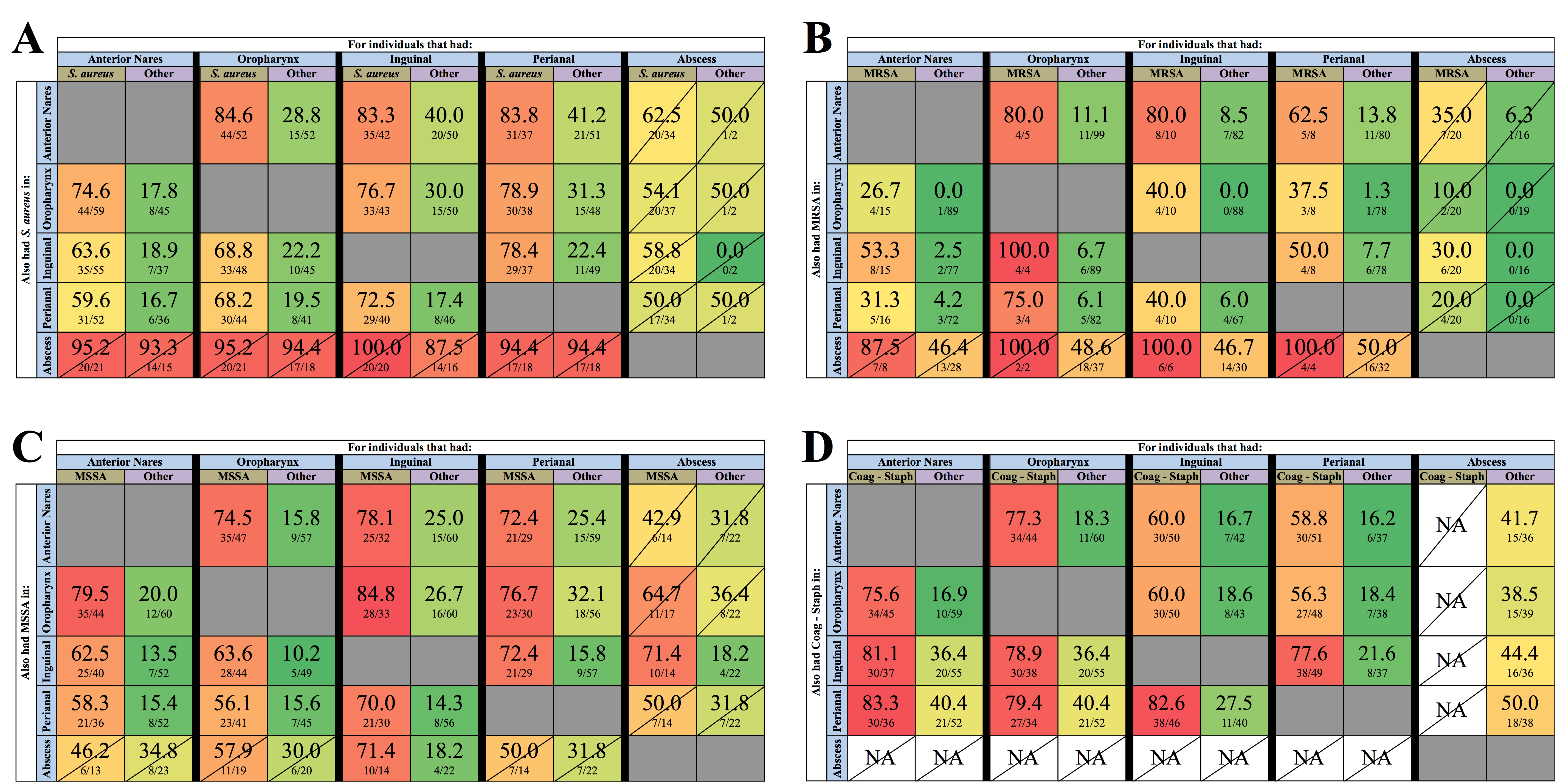

Supplement: Figure S1 [file sph005162157sf1.tif]

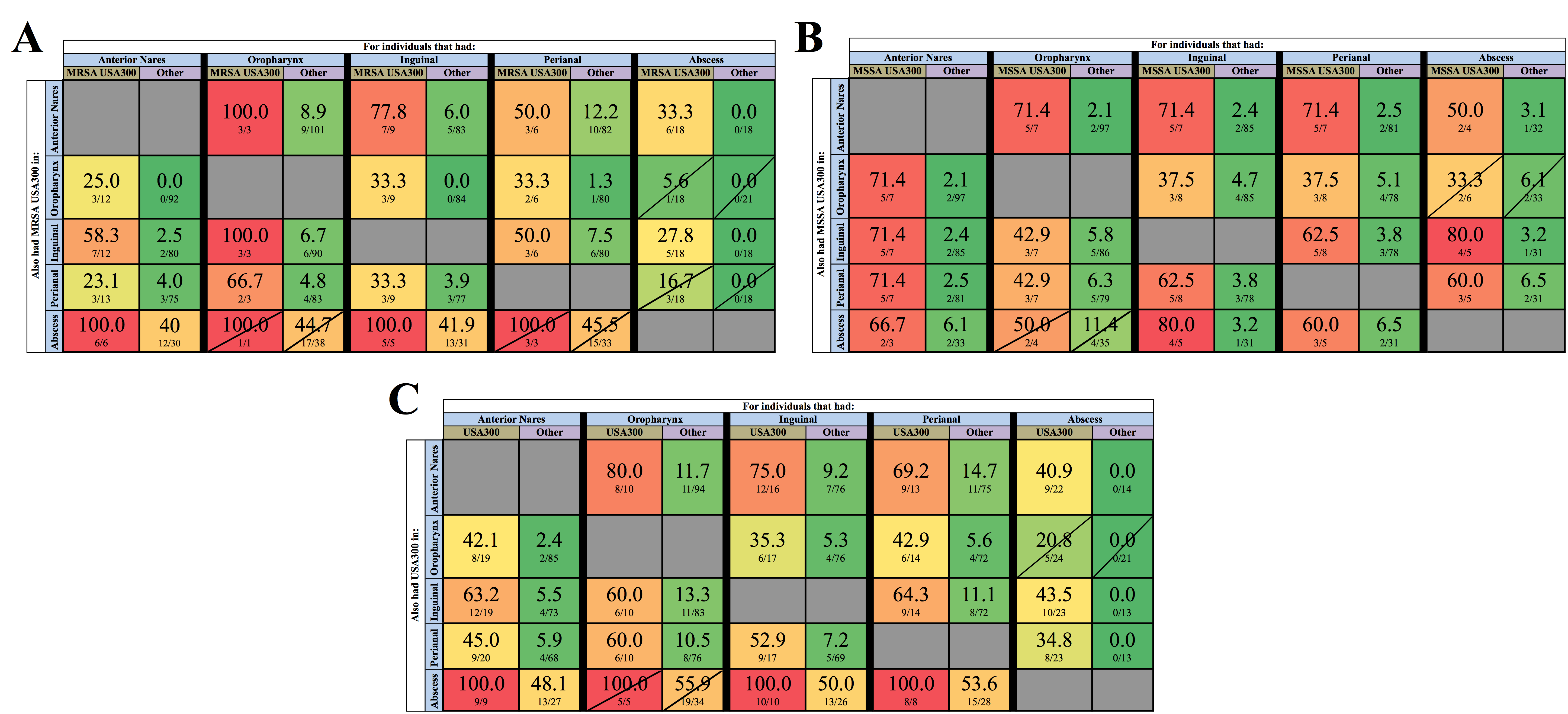

Supplement: Figure S2 [file sph005162157sf2.tif]

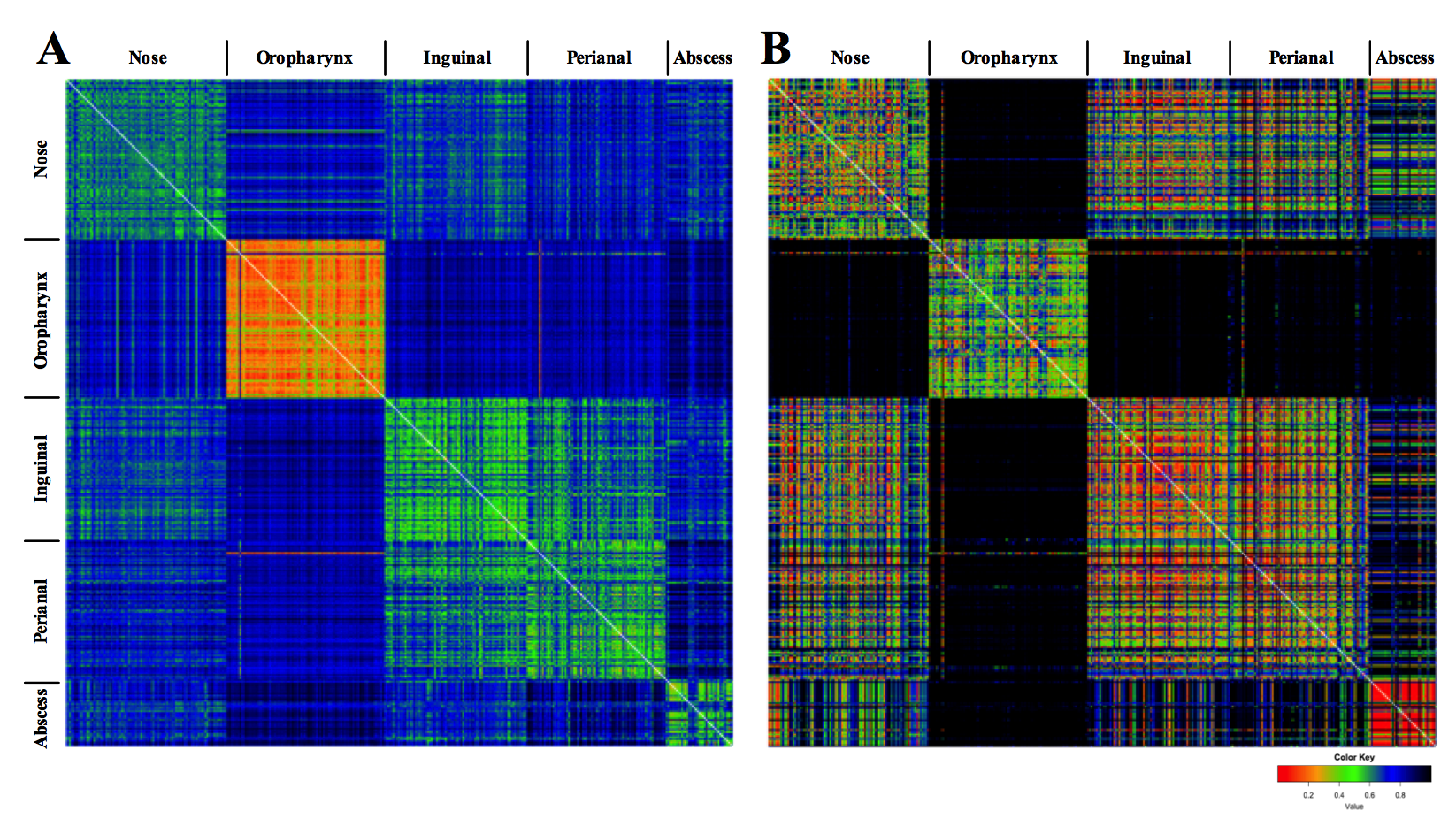

Supplement: Figure S3 [file sph005162157sf3.tif]

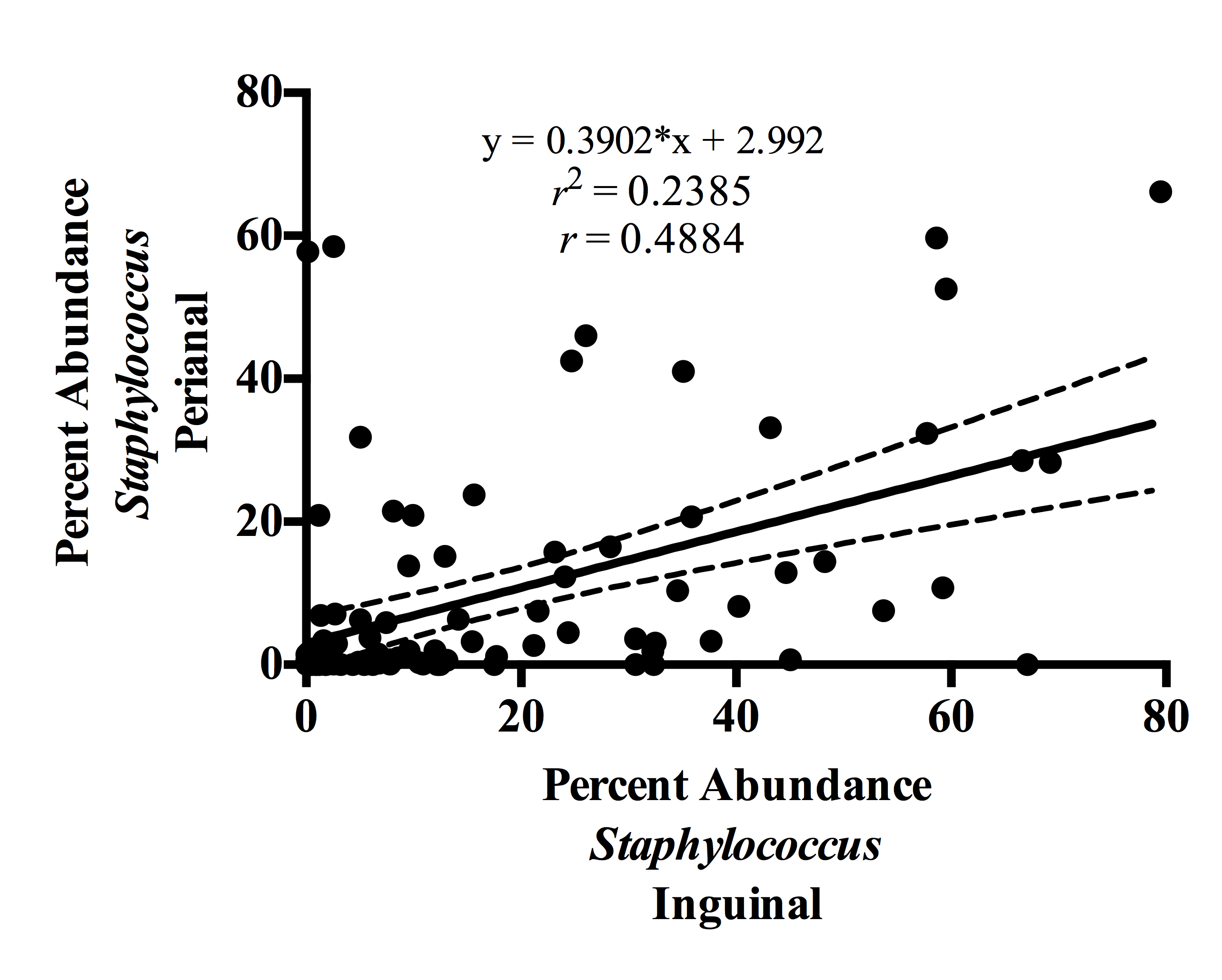

Supplement: Figure S4 [file sph005162157sf4.tif]
